# Benign Cartilaginous Tumors

**Published:** 1860-05

**Authors:** E. Andrews

**Affiliations:** Professor of Surgery in Medical Department Lind University; Chicago


### BENIGN CARTILAGINOUS TUMORS.

By E. ANDREWS, M. D.,
Professor of Surgery in Medical Department Lind University.

Mr. C.——, had a cartilaginous tumor upon the first phalanx of the thumb. When it had attained the size of a black walnut, it was excised by some practitioner, who not being aware that these tumors generally originate from the interior of the bone, simply shaved it off even, and closed up the wound. The consequence was a recurrence of the growth, which in a little time attained its original size. In this state it came under my notice, and I recommended a more thorough extirpation. This was accomplished by laying bare the tumor, cutting off the projecting part, and then gouging out the base which occupied the anterior and interior portions of the shaft of the phalanx. A considerable portion of the bone was thus sacrificed. The wound was then closed and healed by granulation, and the cure was permanent.

Case 2.—Mr. D.——, presented himself with a cartilaginous tumor upon the first phalanx of the index finger, of the size of a large hickory nut. I made an incision through the integuments and laid bare the tumor. It presented a beautiful illustration of the fact that these growths usually originate in the interior of the bone. It had projected from its nidus, carrying before it the periosteum, and derived from that membrane a delicate shell of bone as thin as an egg-shell. Upon removing the tumor, I found the interior to be pure cartilage, subdivided into lobules. The base of the tumor extended into the interior of the bone through an opening, and I was obliged to remove the whole anterior surface of the phalanx, and two-thirds of its thickness, before I could extirpate the cartilage completely. A good deal of inflammation followed, and a large abscess formed in the vicinity, but the result was favorable, and after several months no signs of a return of the tumor were visible.

These tumors generally make their appearance upon the anterior face of the phalanx. Hence, in the operation for removal, the flexor tendons are laid bare, and the wound will seldom unite by first intention; the tendons are entangled in granulations, and adhere to their sheaths. The motions of the second and third joints of the finger, therefore, will be impaired. In view of this result, it is scarcely worth while to recommend very early removal of the growth, unless it be found so situated that the extirpation can be accomplished without exposing the tendons.

